# Intrathecal magnesium sulfate does not reduce the ED_50_ of intrathecal hyperbaric bupivacaine for cesarean delivery in healthy parturients: a prospective, double blinded, randomized dose-response trial using the sequential allocation method

**DOI:** 10.1186/s12871-017-0300-z

**Published:** 2017-01-17

**Authors:** Fei Xiao, Wenping Xu, Ying Feng, Feng Fu, Xiaomin Zhang, Yinfa Zhang, Lizhong Wang, Xinzhong Chen

**Affiliations:** 1 Department of Anesthesia, Women’s Hospital, School of Medicine, Zhejiang University, Hangzhou, China; 2 Department of Anesthesia, Jiaxing Maternity and Child Care Hospital, Jiaxing, Zhejiang China

**Keywords:** Anesthesia, Spinal, Magnesium sulfate, Cesarean delivery

## Abstract

**Background:**

Addition of intrathecal magnesium sulfate to local anesthetics has been reported to potentiate spinal anesthesia and prolong analgesia in parturients. The current study was to determine whether intrathecal magnesium sulfate would reduce the dose of hyperbaric bupivacaine in spinal anesthesia with bupivacaine and sufentanil for cesarean delivery.

**Methods:**

Sixty healthy parturients undergoing scheduled cesarean delivery were randomly assigned to receive spinal anesthesia with 0.5% hyperbaric bupivacaine and 5 μg sufentanil with either 0.9% sodium chloride (Control group) or 50% magnesium sulfate (50 mg) (Magnesium group). Effective anesthesia was defined as a bilateral T_5_ sensory block level achieved within 10 min of intrathecal drug administration and no additional epidural anesthetic was required during surgery. Characteristic of spinal anesthesia and the incidence of side effects were observed. The ED_50_ for both groups was calculated using the Dixon and Massey formula.

**Results:**

There was no significant difference in the ED_50_ of bupivacaine between the Magnesium group and the Control group (4.9 mg *vs* 4.7 mg) (*P* = 0.53). The duration of spinal anesthesia (183 min *vs* 148 min, *P* < 0.001) was longer, the consumption of fentanyl during the first 24 h postoperatively (343 μg *vs* 550 μg, *P* < 0.001) was lower in the Magnesium group than that in the Control group.

**Conclusions:**

Intrathecal magnesium sulfate (50 mg) did not reduce the dose requirement of intrathecal bupivacaine, but can extend the duration of spinal anesthesia with no obvious additional side effects.

**Trial registration:**

This study was registered with Chinese Clinical Trial Registry (ChiCTR) on 15 Jul. 2014 and was given a trial ID number ChiCTR-TRC-14004954.

## Background

Spinal anesthesia is the most widely used technique for cesarean delivery mainly due to its rapid onset and reliable effect [1, 2]. The main limitations of spinal anesthesia are the relatively short duration of anesthesia and analgesia, and high incidence of hypotension. To minimize these limitations, intrathecal adjuncts such as opioids, clonidine, neostigmine and epinephrine have been reportedly used for prolonging analgesia and reducing the dose of intrathecal local anesthetic, and subsequently reducing the incidence of spinal anesthesia-induced hypotension [3–5]. However, intrathecal opioids such as fentanyl and sufentanil, which are commonly used as adjuncts to intrathecal local anesthetic, are associated with a number of undesirable side-effects, including delayed respiratory depression, urinary retention, and pruritus [6–8]. In addition, other adjuncts, such as clonidine, neostigmine and epinephrine, also exhibit adverse effects such as sedation and so on [9, 10].

Magnesium ion is a natural calcium antagonist, which is critical to numerous physiological activities. Animal studies showed that intrathecal magnesium could produce an analgesic effect and enhance opioid’s antinociceptive activity, presumably due to magnesium’s possible block of the N-methyl-D-aspartate (NMDA) receptor and regulate calcium influx into cells in the central nervous system [11, 12]. Several recent studies [13, 14] investigated the utility of magnesium as an adjunct to intrathecal local anesthetics for both obstetrical and nonobstetrical surgery, aiming to overcome the limitations of spinal anesthesia, which main findings are that the addition of magnesium sulfate to intrathecal local anesthetics with or without opioids could prolong the duration of analgesia, reduce postoperative analgesic requirements, and improve perioperative shivering without significant side effects. No previous studies have assessed whether the addition of intrathecal magnesium sulfate can reduce the dose of intrathecal local anesthetic required for spinal anesthesia for cesarean delivery. We therefore designed the present prospective, randomized, double blinded study to investigate the hypothesis that intrathecal magnesium sulfate (MgSO_4_) 50 mg would decrease the median effective dose (ED50, which means the dose that would be necessary to provide effective anesthesia for 50% of the patients treated) of intrathecal hyperbaric bupivacaine in spinal bupivacaine-sufentanil anesthesia for cesarean delivery using an up-down sequential allocation method.

## Methods

### Design

We conducted a prospective, double-blinded, up-down sequential allocation study to determine the ED_50_ of intrathecal hyperbaric bupivacaine combined with or without MgSO_4_, in spinal bupivacaine-sufentanil anesthesia for cesarean delivery in healthy parturients.

### Subjects and setting

Sixty healthy (ASA PS I, II) parturients at term pregnancy, undergoing elective cesarean section, were enrolled in the current study, which was conducted from July 2014 to August 2014. Subjects were enrolled after our hospital’s (Women’s Hospital, School of Medicine, Zhejiang University) ethical review board approval (No: 20140069. Approval date: 2014 Jul 15) and written informed consent have been obtained. Exclusion criteria were patients with obesity (body mass index (BMI) > 35 kg/m^2^), gestational age < 37 weeks, active labor, early labor, ruptured membranes, history of previous cesarean deliveries, diabetes or gestational diabetes, hypertension or pre-eclampsia, intrauterine growth restriction, placenta previa, significant coexisting maternal disease, any contraindication to spinal or epidural anesthesia such as local infection or bleeding disorders. This study was registered in a Chinese Clinical Trial Registry (ChiCTR) (registration number is ChiCTR-TRC-14004954).

### Study protocol

Patients were randomized into one of two groups, Control group (*n* = 30) and Magnesium group (*n* = 30), based on a computer-generated random number list (Microsoft, Excel) which was kept in sealed opaque envelopes before the start of the study (prepared by FX).

No premedication was administered. On arrival in operating theatre, all patients were preloaded with 10 mL · kg^−1^ of 37 °C Lactate Ringer’s solution at the speed of 10 ml · kg^−1^ · h^−1^ with an 18-G intravenous cannula through an arm vein before anesthesia. Standard monitoring including non-invasive blood pressure (NIBP), heart rate (HR), oxygen saturation (SpO_2_) and electrocardiogram (ECG) were applied and recorded.

Combined spinal-epidural (CSE) technique (using the needle-through-needle technique) was performed in the left lateral position for all the patients studied. In brief, epidural puncture was performed with an 18-G Tuohy needle at the estimated L_2-3_ interspace and the method of loss-of-resistance-to-air technique (the air volume is not more than 2 ml) was used to identify the epidural space. A 27-G spinal needle with pencil tip was then passed via the Tuohy needle to enter the subarachnoid space. One of two pre-mixed study solutions was injected at a rate of 0.25 mL · S^−1^through the spinal needle. After the injection, the spinal needle was removed and an epidural catheter was then inserted 3-4 cm into the epidural space. No drugs were injected via the epidural catheter. The patient was then turned to supine with a 15-degree tilt to the left side.

The mixed solutions for spinal anesthesia were prepared before anesthesia by an anesthesia assistant (XZ), who did not participate in the subsequent patient assessment, and administered by a second attending anesthesiologist (FX and WX) who remained blinded to the mixed solution contents. The mixed solution for patients in Control group was: 0.5% bupivacaine + sufentanil 5 μg +0.5 mL 10% dextrose, diluted with 0.9% sodium chloride to a total volume of 3 mL. The mixed solution for patients in Magnesium group was: 0.5% bupivacaine + sufentanil 5 μg + 0.5 ml 10% dextrose + 0.1 ml 50% preservative-free magnesium sulfate (50 mg) (WuXi Pharmaceutical Company, China; Production batch: 1307201.) diluted with 0.9% sodium chloride to a total volume of 3 ml. An insulin syringe (1 ml) was used to measure volumes less than 1 ml.

The dose of intrathecal bupivacaine administered to patients varied according to the up-and-down allocation method [15]. In each group, for the first patient, the dose of intrathecal bupivacaine was 8 mg. For the next patient, the dose of intrathecal bupivacine was determined by the response (effective or ineffective) of the previous patient to the mixed intrathecal solution for spinal anesthesia in the same group. If the response of the previous patient was effective, the dose of intrathecal bupivacaine for the next patient was decreased by 1 mg in that group. Conversely, if the response of the patient was ineffective, the dose of intrathecal bupivacaine for the next patient was increased by 1 mg in that group. Effective anesthesia was defined as a bilateral T_5_ or above sensory block level achieved within 10 min of intrathecal drug administration and no additional epidural anesthetic was required for intraoperative pain. Ineffective anesthesia was defined as a bilateral T_5_ sensory block level was not achieved within 10 min of intrathecal drug administration, or an additional epidural anesthetic was needed to deal with intraoperative pain (VAS ≥ 3) despite a T_5_ sensory level being obtained. Additional epidural anesthetic was 5 ml of 2% lidocaine, repeated every 10 min if necessary.

### Measurements

Automatic measurements of non-invasive arterial pressure (NIBP) and heart rate (HR) were recorded from the beginning of spinal anesthesia at 2-min intervals for 10 min, and then at 5-min intervals until the end of the surgery. An average of three consecutive measurements at the time when patient arrived in operating theatre with a supine position was defined as basal NIBP and basal HR. Hypotension was defined as a systolic arterial pressure below 90 mmHg, or a decrease of more than 20% of basal systolic blood pressure. Hypotension was treated with a boluse of 40 μg intravenous phenylephrine, repeatedly if needed. Bradycardia, defined as heart rate less than 55 beats per min, was treated with 0.5 mg of atropine intravenously.

Sensory level was assessed bilaterally along the mid clavicular line using a 17-G needle (patient was asked to report pain sensation, if the block was not even bilaterally, the lower side was chosen). The onset time of sensory block was defined as the time between intrathecal injection and a T_10_ sensory block level being achieved. The duration of sensory block was defined as the time between the onset time of sensory block and the recovery of sensory level of T_10_. Motor block in the lower limbs was graded by a Bromage Score [16] (0 = able to lift extended leg; 1 = able to flex knee but not lift extended leg; 2 = able to move foot only; and 3 = unable to move foot). The onset time of motor block was defined as the time between intrathecal injection and a Bromage Score of 1 being reached. The duration of motor block was defined as the period between the time of motor block onset and a Bromage Score of 0. The duration of spinal anesthesia [17] was defined as the period from spinal injection to the first requirement of bolus of fentanyl 10 μg postoperatively with patient-controlled analgesia (PCA) pump, which was set with a bolus of 10 μg fentanyl and 10 min of locking time and without a background dose. And patient did not received any other analgesics after surgery Both the sensory and motor block characteristics were noted every 1 min for the first 10 min after spinal anesthesia, followed thereafter by 10-min intervals until the end of the surgery and then by 30-min intervals after surgery before the patient full recovery.

Subjective pain was assessed with a visual analogue scale (VAS) ranged from 0 to 10 (0 = no pain, 10 = maximum undersirable pain) at the following timepoints: skin incision, fetal delivery, peritoneal closure, skin closure, and 1, 4, 8, 12, 24 h postoperatively. At the end of the surgery, patients were asked to grade the level of satisfaction during surgery (1 = excellent; 2 = good; 3 = bad).

Side effects and complications of spinal anesthesia including pruritus, shivering, severe sedation, nausea and vomiting, post dural puncture headache (PDPH) and respiratory depression (defined as breath rate < 12 bpm or SpO2 < 90%) during surgery and the first 24 h postoperatively were also recorded by a fixed anesthesia assistant. Sedation was ranked as none = awake and alert, mild = awake but drowsy, moderate = asleep but arousable, severe = not arousable. Any symptoms and signs of neurological deficit were also recorded. Umbilical arterial blood was drawn for blood gas analysis immediately after delivery. The neonatal Apgar score was assessed at 1 min and 5 min after delivery by a pediatrician who was not involved in this study.

### Statistical analysis

The Dixon and Massey formula [15, 18] was applied to calculate the ED_50_ for both groups. Sample size estimation was calculated using the G*Power software. The primary outcome of the present study which is ED_50_ of intrathecal bupivacaine for cesarean delivery. An estimated ‘average’ SD of difference of the ED_50_ of intrathecal bupivacaine between groups is 0.5 mg, and power was given at 0.95 to detect a difference of 1.6 SD (0.8 mg) at *P* < 0.05. A minimum of 12 subjects was then necessary in each of the two groups. Because the Dixon and Massey technique requires the sample size to be approximately twice this number (as the estimations of ED50, SE and confidence interval (CI) 95% are based on the number and distribution of the lesser occurring outcome, which will be approximately 50% of the observations), therefore, 30 subjects were enrolled finally in each of the two groups, allowing for possible drop-outs and a potential deviation of the initial dose from the center of the effective dose distribution.

Demographic data were collected and are presented as count or mean ± SD as appropriate. Nominal data were analyzed using the Chi-square test, normally distributed continuous data were analyzed using Student’s *t* test and non-normallly distributed continuous data (such as epidural supplementations which were presented as median) were analyzed using non-parametric Wilcoxon rank sum test. Normal distribution was determined using the Kolmogorov–Smirnov test. Duration of spinal anesthesia was also analyzed using Kaplan-Meier survival analysis. Statistical analysis was performed with Graphpad Prism 5 (Version 5.01). Statistical significance was defined as *P* < 0.05 (two-sided).

## Results

**Fig. 1:**
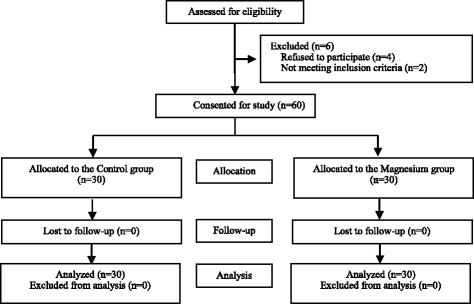
CONSORT diagram

The CONSORT diagram of the present study is showed in Fig. 1. A total of 66 patients were assessed for eligibility, among them 60 patients were enrolled and randomly assigned into the Control group (*n* = 30) or the Magnesium group (*n* = 30). All 60 patients finished the study and were included into the final analysis.

There were no significant differences in the demographic and obstetric characteristics between the Control group and the Magnesium group (Table 1).

**Table 1:** Patient’s demographic, obstetric and surgical data

	Magnesium group (*n* = 30)	Control group (*n* = 30)	*P*-value*
Age (y)	25 ± 3	26 ± 3	0.41
Height (cm)	162 ± 3	162 ± 3	0.42
Weight (kg)	72 ± 4	72 ± 4	0.84
Gestational age (week)	39 ± 1	39 ± 1	0.60
Duration of surgery (min)	45 ± 7	47 ± 7	0.41

Data are presented as mean ± SD. *Student *t* test

**Fig. 2:**
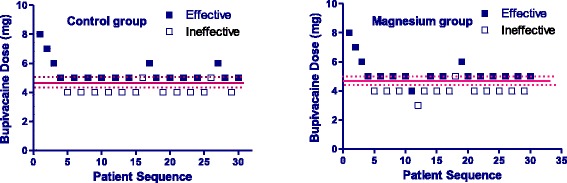
Individual response to intrathecal hyperbaric bupivacaine at corresponding dose. Unfilled square (□) represents an ineffective response to the corresponding dose of intrathecal bupivacaine for spinal anesthesia. Filled square (■) represents an effective response to the corresponding dose of intrathecal bupivacaine for spinal anesthesia. Solid line represents the ED50 (dashed lines represent the 95% confidence interval, CI) of intrathecal hyperbaric bupivacaine for caesarean delivery

The ED_50_ of intrathecal hyperbaric bupivacaine for cesarean delivery, determined using Dixon and Massay up-down sequential method [19, 20], was 4.7 mg (95% CI, 4.4–5.0 mg) in the Control group, and 4.9 mg (95% CI, 4.6–5.2 mg) in the Magnesium group. There was no significant difference in the ED_50_ of bupivacaine between the Magnesium group and the Control group (*P* = 0.53). The individual responses (effective or ineffective anesthesia) to the corresponding intrathecal hyperbaric bupivacaine dose are showed in Fig. 2. Thirteen patients in each group required additional epidural 2% lidocaine to complement intra-operative analgesia,and the mean total dose of additional epidural 2% lidocaine was similar in the two groups [5 ml (5–10 ml) vs. 5 ml (5–10 ml)].

**Fig. 3:**
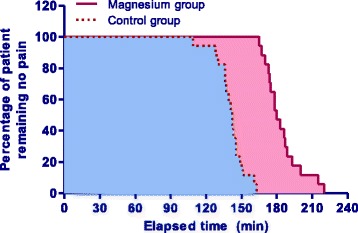
Duration of spinal anesthesia. Cumulative percentages of patient remaining no pain after spinal injection in patients with “effective anesthesia” in the Magnesium group (*solid line*, *red area*) and in the Control group (*dotted line*, *blue area*), obtained using the Kaplan–Meier survival analysis. Log-rank differences between the two groups were significant (*P* < 0.001)

Characteristics and efficacy of spinal anesthesia in patients with “effective anesthesia” are presented in Table 2. The onset and duration of sensory and motor blockade were longer in the Magnesium group than in the Control group (*P* < 0.001). Moreover, the duration of spinal anesthesia was also significantly longer in the Magnesium group than in the Control group (183 ± 11 min *vs* 148 ± 9 min, *P* < 0.001) (Fig.3). The consumption of fentanyl during the first 24 hours postoperatively were significantly less in the Magnesium group than in Control group (343 ± 42 μg *vs* 550 ± 49 μg, *P* < 0.001). The Magnesium group has higher rate of excellent satisfaction during intraoperative period than that in the Control group (94.1% *vs* 52.9%, *P* = 0.017).

**Table 2:** Characteristics and efficacy of spinal anesthesia in patients with effective anesthesia

	Magnesium group (*n* = 17)	Control group (*n* = 17)	*P*-value
Sensory block (to pinprick)			
Onset time to T_10_ (min)	4 ± 0	3 ± 0	<0.001*
Duration (min)	140 ± 9	121 ± 9	<0.001*
Motor block			
Onset time (min)	4 ± 0	2 ± 0	<0.001*
Duration (min)	148 ± 12	125 ± 10	<0.001*
Duration of anesthesia (min)	183 ± 11	148 ± 9	<0.001*
Consumption of fentanyl (μg)	343 ± 42	550 ± 49	<0.001*
Patient Satisfaction			
Excellent [number (%)]	16 (94.1)	9 (52.9)	0.017^#^
Good [number (%)]	1 (5.9)	8 (47.1)	0.017^#^

Data are presented as mean ± SD or number (%). *Student *t* test, ^#^Chi-square test

The incidence of side effects of spinal anesthesia, such as hypotension, nausea and vomiting, shivering, pruritus, post dural puncture headache (PDPH), severe sedation and respiratory depression during perioperative period, were similar between groups (Table 3). Neonatal Apgar score at 5 min after delivery and umbilical arterial pH immediately after delivery were also comparable between groups (Table 3). No neurological deficit was observed in any patient in both groups during the first postoperative week.

**Table 3:** Side effects of anesthesia and neonatal Apgar score and umbilical arterial pH

	Magnesium group (*n* = 30)	Control group (*n* = 30)	*P* -Value
Hypotension	6(20.0)	8(26.7)	0.76^#^
Nausea and vomiting	12(40.0)	9(30.0)	0.59^#^
Shivering	5 (16.7)	6 (20.0)	1.00^#^
Pruritus	8(26.7)	7(23.3)	1.00^#^
PDPH	0 (0%)	1 (3.3)	1.00^#^
Severe sedation	0	0	
Respiratory depression	0	0	
Apgar score	10.0 ± 0.0	10.0 ± 0.0	1.00*
Umbilical artery pH	7.37 ± 0.04	7.38 ± 0.06	0.22*

Data are presented as number (percent) or mean ± SD. PPDH = post dural puncture headache. *Student *t* test, ^#^Chi-square test

## Discussion

We demonstrated that intrathecal magnesium sulfate (50 mg) did not reduce the median effective dose (ED_50_) of intrathecal bupivacaine, for cesarean delivery under spinal anesthesia with bupivacaine coadministered with 5 μg sufentanil in healthy parturients.

Several previous studies [13, 19, 21, 22] reported that the duration of spinal anesthesia was significantly prolonged by intrathecal magnesium sulfate, which is consistent with the findings in the present study. The present study also showed that adding magnesium sulfate intrathecally could significantly prolong the duration of spinal anesthesia with bupivacaine and sufentanil (184 min *vs* 148 min, *P* < 0.001). Evidence is conflicting regarding the usage of intrathecal magnesium sulfate in obstetric patients for prolonging the duration of spinal anesthesia [13, 17, 22, 23], the study designs with or without opioids may contribute to this discrepancy. This synergistic effect has been already demonstrated in a rat model by Kroin and colleagues [12] who found that the addition of intrathecal magnesium increased the peak effect and area under the analgesic curve of intrathecal morphine. The potentiation of opioid antinociception by magnesium sulfate may last in the postoperative period, explaining the decrease in consumption of postoperative fentanyl found in the present study.

NMDA-receptor antagonists can diminish the activation of C-fibers which leads to neuronal excitation, prevent central sensitization elicited by peripheral nociceptive stimulation [20, 24]. Magnesium sulfate, a noncompetitive NMDA-receptor antagonist, has both independent and synergistic analgesic properties. Kroin et al. demonstrated in an animal study that intrathecal magnesium sulfate potentiated the antinociceptive effect of morphine to noxious thermal and mechanical stimulation at an incisional pain site at the level of the spinal cord in a dose-dependent fashion [12]. Mercieri et al. found that systemic magnesium sulfate infusion (i.e. intravenous route), even with large doses, did not increase cerebrospinal fluid (CSF) magnesium concentrations, suggesting magnesium sulfate exhibits insufficient blood-brain barrier penetration [25, 26]. Hence, intrathecal route would be better for magnesium sulfate administration to potentiate spinal anesthesia than systemic route by which effective CSF concentrations of magnesium required large doses that may result in severe side effects.

Because intrathecal magnesium alone has been showed to produce sensory and motor block, [27, 28] it might be expected that magnesium potentiates the spinal block via a synergistic interaction between NMDA antagonists and local anesthetics, resulting in a reduction in the dose of local anesthetics required for achieving effective spinal anesthesia for certain surgical procedures. Unexpectedly, the present study demonstrated that the ED_50_ of intrathecal bupivacaine for cesarean delivery in the Magnesium group was not reduced when compared with the Control group, suggesting that intrathecal 50 mg magnesium sulfate exhibits little or no effect on efficacy of spinal anesthesia with local anesthetics for cesarean delivery. In contrast to the lack of effect of magnesium on the median effective dose of intrathecal bupivacaine in the current study, previous studies suggested that intrathecal fentanyl or sufentanil significantly reduce the dose (ED_50_ or ED_95_) of spinal local anesthetics for cesarean delivery [3, 29, 30]. The possible underlying mechanism is that magnesium may be removed from extracellular fluid more rapidly than opioids, or that it may be specific to the NMDA receptor channel and therefore has no influence on the channels the local anesthetics act and opioid receptor sites [14, 17]. Moreover, intrathecal magnesium sulfate exerts its spinal action in a localized manner, [17] whereas, fentanyl or sufentanil bind strongly to opioid receptors in the dorsal horn of spinal cord, and may also exert a supraspinal action by intrathecal cephalad spread, [31] hence both fentanyl and sufentanil exhibit a significant synergistic effect on local anesthetics. In addition, the dosage of intrathecal magnesium sulfate should be taken into account. The dose of magnesium sulfate of 50 mg we choose in the current study was based on majority of the studies [13, 14, 17, 32] on clinical investigation of intrathecal magnesium sulfate for cesarean delivery publically published so far. However, whether higher dose of intrathecal magnesium sulfate could reduce the dose (ED_50_ or ED_95_) of intrathecal local anesthetics for cesarean delivery remains unknown. Hence, it is warrant to conduct further studies on optimal dose of magnesium sulfate for cesarean delivery.

The onset of sensory and motor blockade in the Magnesium group in the present study were found to be significantly delayed when compared with the Control group, which was in agreement with the findings of previous studies [13, 21]. The clinical significance of this delay is questionable because the delayed time was only about 1 min for both sensory and motor blockade onset in the present study. It is difficult to explain this phenomenon on mechanism of magnesium action upon central nervous system. The effect of adding magnesium sulfate on the pH and baricity of the spinal solution might be considered as a possibility for this delay [22, 33]. Pascual-Ramirez suggested that the onset delay when magnesium was added could also indicate there is a modulation of the neuronal electrical conduction blockade [34].

Concerns about the safety of intrathecal administration of magnesium sulfate have been being considered. Preclinical studies showed the impact of intrathecal magnesium sulfate on neurological structure and functions appears inconsistent among species [33]. In rats, intrathecal magnesium sulfate resulted in transient motor and sensory block with no obvious adverse clinical and histological consequences. In canines, intrathecal magnesium sulfate of 45–60 mg produced no neurological deficit and histopathological change in spinal cord [35]. In clinical studies, intrathecal magnesium sulfate 50 mg was found to be safe and effective, [13, 14, 17, 21, 22] which are similar to the findings of the present study, in which we also did not find any obvious symptoms and signs of dysfunction in nervous system, reinforcing the safety of maternal intrathecal magnesium. However, safety of intrathecal magnesium sulfate would be argued because our study is a small study and no specific assessments to assess safety were done. Hence, the safety of intrathecal magnesium sulfate with larger sample size and specific assessment variables, or with large dose should be carefully evaluated in both animals and humans, especially in pregnant populations.

## Conclusions

In conclusion, in patients undergoing cesarean delivery with spinal anesthesia, the addition of intrathecal magnesium sulfate (50 mg) to spinal hyperbaric bupivacaine combined with sufentanil did not reduce the ED_50_ of intrathecal bupivacaine as determined with an up-down sequential method, but prolonged the duration of spinal anesthesia, reduced the consumption of post-operative fentanyl, delayed the onset of both sensory and motor blockade of spinal anesthesia. No obvious additional side effects were found.

## Abbreviations

BMI: Body mass index
ECG: Electrocardiograph
ED_50_: Median effective dose
HR: Heart rate
MgSO_4_: Magnesium sulfate
NIBP: Non-invasive blood pressure
NMDA: N-methyl-D-aspartate
PCA: Patient-controlled analgesia
PDPH: Post dural puncture headache
SpO_2_: Pulse oxygen saturation
VAS: Visual analogue scale
